# ﻿A new species of *Tetragoniceps* Brady, 1880 (Copepoda, Harpacticoida, Tetragonicipitidae) from an anchialine cave in Bermuda, with an updated key to the species of the genus

**DOI:** 10.3897/zookeys.1239.144436

**Published:** 2025-05-20

**Authors:** Giovanni Mussini, Yuuki J. Niimi, Sahar Khodami, Terue C. Kihara, Pedro Martinez Arbizu, Leocadio Blanco-Bercial

**Affiliations:** 1 Department of Earth Sciences, University of Cambridge, Downing St., Cambridge CB2 3EQ, UK University of Cambridge Cambridge United Kingdom; 2 Bermuda Institute of Ocean Sciences (BIOS), Hamilton St. George’s, Bermuda Bermuda Institute of Ocean Sciences (BIOS) Hamilton St. George’s Bermuda; 3 Senckenberg am Meer, German Center for Marine Biodiversity Research, Südstrand 44, 26382, Wilhelmshaven, Germany Senckenberg am Meer, German Center for Marine Biodiversity Research Wilhelmshaven Germany

**Keywords:** Bermuda, cave fauna, copepods, Crustacea, endemism

## Abstract

*Tetragonicepsbermudensis***sp. nov.** (Copepoda, Harpacticoida, Tetragonicipitidae) is described based on an ovigerous female collected from Roadside Cave, a tidally influenced anchialine cavern in Bermuda. The new taxon represents the 16^th^ recorded species of *Tetragoniceps* Brady, 1880. It can be distinguished from congeneric species by the length:width ratio of the caudal rami (approximately 10 times longer than wide), its cephalothorax with a smooth dorsal surface, and the diagnostic setal formula of its pereiopods 1–5. *Tetragonicepsbermudensis***sp. nov.** is the first record of *Tetragoniceps* from Bermuda and the first known anchialine species in the genus globally. Based on our description of the new species, we provide a revised key to the species of *Tetragoniceps*. In addition, we include an updated table of salient morphological characters for the females of the genus, providing grounds for a preliminary analysis of the phylogenetic relationships of its constituent species.

## ﻿Introduction

Bermuda’s Walsingham cave system is a global hotspot of anchialine biodiversity (Iliffe and Calderón-Gutiérrez 2021). This network of karstic limestone caverns hosts at least 79 native anchialine species, including 67 crustaceans. Of these, 21 species are copepods (Iliffe and Calderón-Gutiérrez 2021; Varela et al. 2023). Their distribution across 15 genera denotes multiple independent colonisation events since the Pleistocene formation of Bermuda’s anchialine cave system, most likely from deepwater crevicular habitats in the island’s volcanic bedrock (van Hengstum et al. 2019; Iliffe and Calderón-Gutiérrez 2021).

A significant portion of the world’s anchialine copepod diversity is represented by the cosmopolitan order Harpacticoida Sars, 1903 (Dole-Olivier et al. 2000; Varela et al. 2023). Fifteen harpacticoid species from eight genera and six families are known to inhabit anchialine environments, and five of these are found in the limestone caves of Bermuda (Varela et al. 2023). Except for the recently described *Eupeltehughesi* Varela, Illiffe & Walter, 2023 (Peltidiidae Claus, 1860), all known Bermudian anchialine harpacticoids belong to the family Superornatiremidae Huys, 1996 (Varela et al. 2023). Despite being very speciose in the tropics (Boxshall and Halsey 2004) and occurring in the waters around Bermuda (Fiers 1995), the family Tetragonicipitidae Lang, 1944, which comprises 12 genera globally (Björnberg and Kihara 2013), has never been recorded in the island’s caves. The only known anchialine tetragonicipitid, belonging to the genus *Phyllopodopsyllus* T. Scott, 1906 is found in groundwaters from Western Australia (Karanovic et al. 2001).

We provide the first record of Tetragonicipitidae in Bermuda’s anchialine caves by describing a new species of *Tetragoniceps* Brady, 1880. The genus comprises 15 previously described valid species: *T.malleolatus* Brady, 1880, *T.dubia* Thompson & A. Scott, 1903, *T.scotti* Sars, 1911, *T.brevicauda* T. Scott, 1900, *T.truncata* Nicholls, 1940, *T.longicaudata* Nicholls, 1940, *T.arenicolus* Krishnaswamy, 1957, *T.bergensis* Por, 1965, *T.brownei* Wells, 1967, *T.prima* (Coull, 1971), *T.bookhouti* Coull, 1971, *T.unguis* Wells & Rao, 1987, *T.galapagoensis* Mielke, 1989, *T.santacruzensis* Mielke, 1997, and *T.pacificus* Burgess, 1998. The new taxon described here represents the first known anchialine species of *Tetragoniceps*, and the first record of the genus in Bermuda.

## ﻿Material and methods

### ﻿Specimen collection

**Figure 1.**
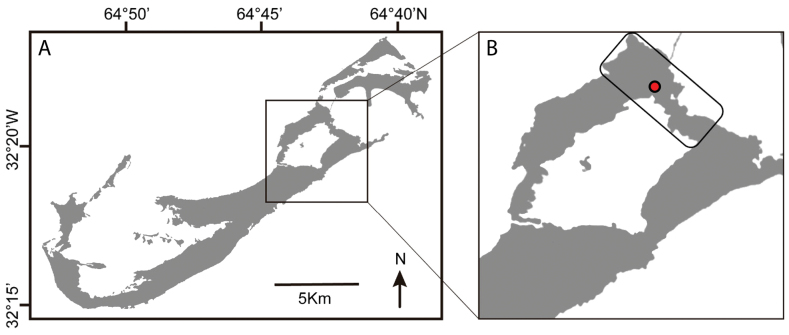
Map showing the location of Roadside cave, the type locality of *Tetragonicepsbermudensis* sp. nov. **A** island of Bermuda **B** detail of Bermuda’s Harrington Sound area, with rectangle highlighting the Walsingham karst region. The approximate location of Roadside cave is indicated by the red dot.

**Figure 2.**
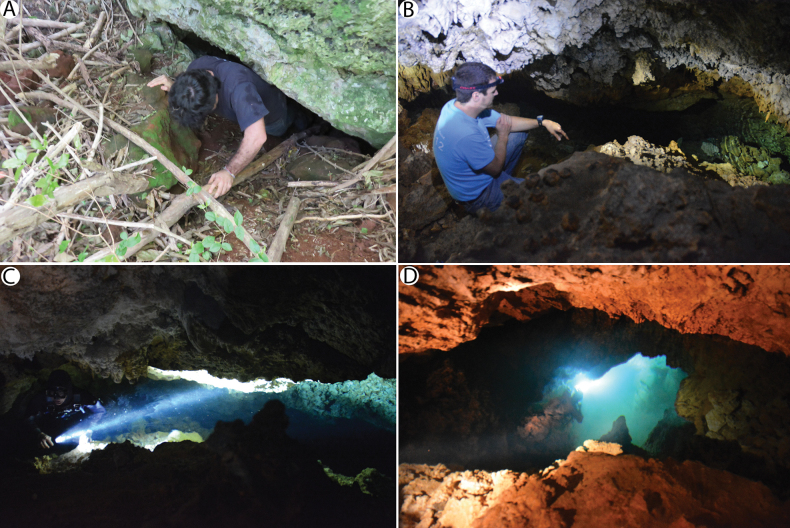
Collection site at Roadside Cave, Walsingham District, Bermuda **A** entrance at the base of a rock outcrop **B** cave fissure, extending into **C** narrow passageway leading to **D** tidal pool.

The type and only known specimen, an adult ovigerous female (USNM 1752730), was collected from Roadside Cave, a small anchialine cavern in the Walsingham District of Bermuda (Fig. 1) located approximately 110 m from the nearest shore at Harrington Sound. Roadside Cave contains a tidal pool about 8 m deep and 1 m wide, which follows a fissure blocked at the bottom by breakdown rubble and is accessible through a hole at the base of a rock outcrop (Fig. 2). The pool has a tidal range approximately 57% that of the open sea, with a lag averaging 71 min. Salinities have been reported as 30.2‰ at the surface and 31.8‰ at 1 m (Bowman and Iliffe 1985; Fosshagen and Iliffe 1988).

Plankton collections were made with a 25 cm diameter, 50 µm mesh size net, on 5 April 2016 in Roadside Cave. The net was trawled near the bottom of the pool by a diver. Samples were kept in glass jars, alive, until identification under a dissecting microscope. Individuals were then fixed in DESS following Yoder et al. (2006). DNA was extracted from the whole individual using a 25 µL Chelex extraction, as described by Estoup et al. (1996); however, the sequences of the studied *Tetragoniceps* specimen were found to be too low in quality (mixed peaks) for further study. The remaining exoskeleton was then transferred to glycerine on a glass slide and stored in alcohol as a voucher for morphological description.

### ﻿Confocal laser scanning microscopy (CLSM)

After Chelex DNA extraction, the specimen was stained overnight with a 1:1 solution of Congo Red and Acid Fuchsin using procedures adapted from Michels and Büntzow (2010). The whole specimen was temporarily mounted onto a slide with glycerine, and self-adhesive plastic reinforcement rings were used to support the coverslip, as detailed by Kihara and da Rocha (2009).

Imaging was performed using a Leica TCS SP5 laser scanning microscope (Suppl. material 1) equipped with a Leica DM5000 B upright microscope and three visible-light lasers (DPSS 10 mW 561 nm; HeNe 10 mW 633 nm; Ar 100 mW 458, 476, 488 and 514 nm), combined with the LAS AF software v. 2.2.1. (Leica Application Suite Advanced Fluorescence). Images were obtained using the objectives HCX PL APO CS 10.0 × 0.40 DRY UV and HCX APO U-V-I 40.0 × 0.75 DRY UV, a 561 nm excitation wavelength with an 80% acousto-optic tuneable filter (AOTF).

Series of stacks were obtained, collecting overlapping optical sections throughout the whole preparation with an optimal number of sections according to the software. The acquisition resolution was 2048 × 2048 pixels, final images were obtained by maximum projection, and CLSM illustrations were composed and adjusted for contrast and brightness using Adobe Photoshop CS5.

### ﻿Photography

Images for the morphological drawings, made in Adobe Photoshop 2024, were taken using an Olympus IX-83 inverted microscope, using multiple objectives (20× to 100×) to capture the full habitus of the holotype and the details of the appendages, and checked for consistency with microscopy observations and confocal images at various magnifications. Individual photographs were stacked by layers to ensure that morphological characters were properly imaged.

### ﻿Phylogenetic analysis

To test the interrelationships of the new taxon and congeneric species, an exploratory maximum-parsimony (MP) phylogenetic analysis of *Tetragoniceps* was performed in TNT v. 1.5 (Goloboff et al. 2008). The analysis was based on a morphological character matrix including all known females of *Tetragoniceps* (Data S1) and based on the salient characters for the genus listed by Coull (1973). *Protogonicepshebraeus* Por, 1964 was chosen as the outgroup. *Protogoniceps* is a monospecific genus within Tetragonicipitidae, sharing with *Tetragoniceps* key characters including the presence of an unguiform antennular projection, a first antennular segment at least 2.5 times as long as the second, and a non-foliaceous female P_5_ (Fiers 1995). Moreover, *Protogoniceps* was regarded by Por (1964) as having a morphology close to that of the common ancestor of Tetragonicipitidae based on its mosaic of traits found in disparate genera within the family: a rostrum and swimming leg armatures similar to those of *Pteropsyllus* T. Scott, 1906, a spur located on the second antennular segment comparable to that of *Phyllopodopsyllus* T. Scott, 1906, and 9-segmented antennules also found in *Tetragoniceps* (Por 1964).

MP analyses were carried out using the Traditional Search option under equal weights (EW) and default settings (random seed = 1, 10 replicates, and using a tree bisection reconnection algorithm). The four most parsimonious trees were collapsed into a strict consensus topology. Resampling (100 replicates) was then conducted through standard bootstrapping (sampling with replacement) and jackknifing using a default removal probability of 36.

### ﻿Descriptive abbreviations

**A_1_**, antennule; **A_2_**, antenna; **ae**, aesthetasc; **benp.**, baseoendopodite; **enp.**, endopodite; **exp.**, exopodite; **P_1_–P_6_**, pereiopods 1–6.

## ﻿Results

### ﻿Taxonomic description

**Order Harpacticoida Sars, 1903**

**Family Tetragonicipitidae Lang, 1944**

#### ﻿Genus Tetragoniceps

Taxon classificationAnimaliaHarpacticoidaTetragonicipitidae

Brady, 1880

EC0F016E-A4F9-5FEC-88C0-81EEA48C028C

##### Genus diagnosis (modified from Coull 1973).

Caudal rami variable, ranging from as wide as long to approximately 10 times longer than wide. Body subcylindrical. Rostrum small or absent. A_1_ female 8- or 9-segmented, with first segment elongate, and with dentiform projection pointing medially or laterally. Exopodite A_2_ well developed, with 2 or 3 setae. Endopodite of maxillule well developed. Maxilla with 3 to 5 endites, and with allobasis bearing a claw-like spine. Mandible bearing *pars incisiva* with dentations, with both exopod and endopod 1- or 2-segmented. Maxilliped subchelate, with basis bearing no more than 2 setae. P_1_ to P_4_ with 2-segmented endopodites and 3-segmented exopodites; setal formulae variable. Female P_5_ confluent or distinct. Setation on female P_5_ variable. Where known, male P_5_ distinct. Sexual dimorphism in P_2_ or P_2_ and P_3_, or caudal rami. Male A_1_ 7- to 9-segmented, with or without dentiform projection.

##### Type species.

*T.malleolatus* (Brady, 1880)

##### Other species.

*T.dubia* Thompson & A. Scott, 1903, *T.scotti* Sars, 1911, *T.brevicauda* T. Scott, 1900, *T.truncata* Nicholls, 1940, *T.longicaudata* Nicholls, 1940, *T.arenicolus* Krishnaswamy, 1957, *T.bergensis* Por, 1965, *T.brownei* Wells, 1967, *T.prima* (Coull, 1971), *T.bookhouti* Coull, 1971, *T.unguis* Wells & Rao, 1987, *T.galapagoensis* Mielke, 1989, *T.santacruzensis* Mielke, 1997, *T.pacificus* Burgess, 1998.

#### ﻿ Tetragoniceps bermudensis sp. nov.

Taxon classificationAnimaliaHarpacticoidaTetragonicipitidae

90289239-F1DC-56D1-8E37-B2CB5B9154D9

https://zoobank.org/8004F50D-6530-460E-AFAF-EB9B6F7EBFF6

##### Type locality.

Bermuda, Roadside Cave (32.3468, −64.7131).

##### Type material.

Holotype, ovigerous female bearing egg sac. National Museum of Natural History (USNM 1752730).

##### Etymology.

The specific name *bermudensis* refers to the place where the new species was found followed by the Latin suffix -*ensis*, i.e. living in, or coming from, Bermuda. It is an adjective in the nominative singular.

##### Diagnosis.

A *Tetragoniceps* with caudal rami approximately 10 times longer than wide, P_5_ with baseoendopodite and exopodite not fused into a single plate, cephalothorax with smooth dorsal surface, last segment of exopodites of P_2_ to P_4_ with 1 inner seta, endopodites of P_2_ to P_4_ with setal formula 1.021, and P_5_ exopodite with 6 setae.

##### Description.

**Figure 3.**
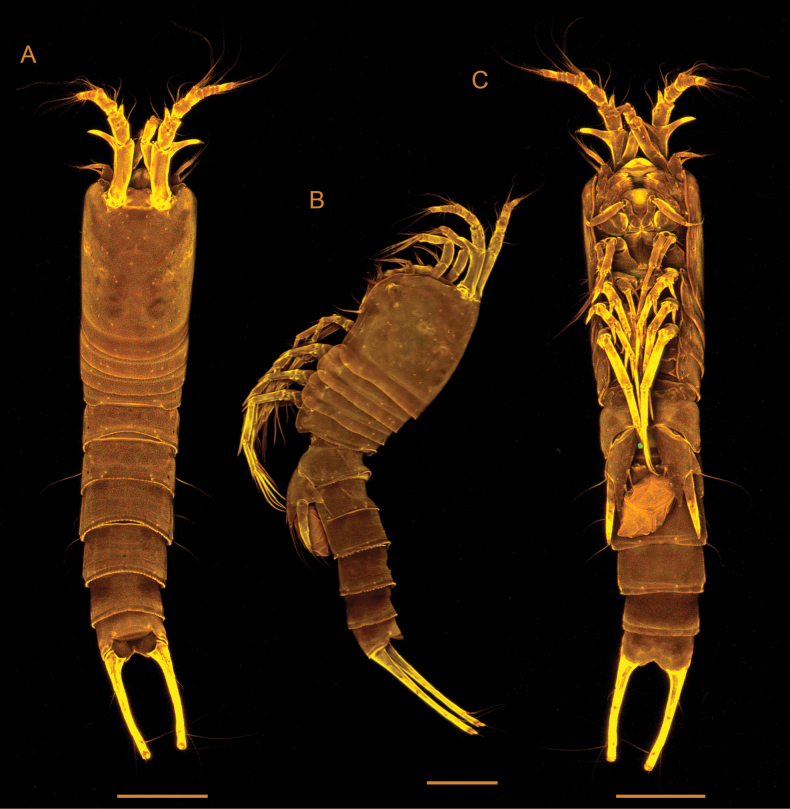
*Tetragonicepsbermudensis* sp. nov., female. Confocal laser scanning microscopy images **A** habitus, in dorsal view **B** habitus, in lateral view **C** habitus, in ventral view. Scale bars: 100 µm.

**Figure 4.**
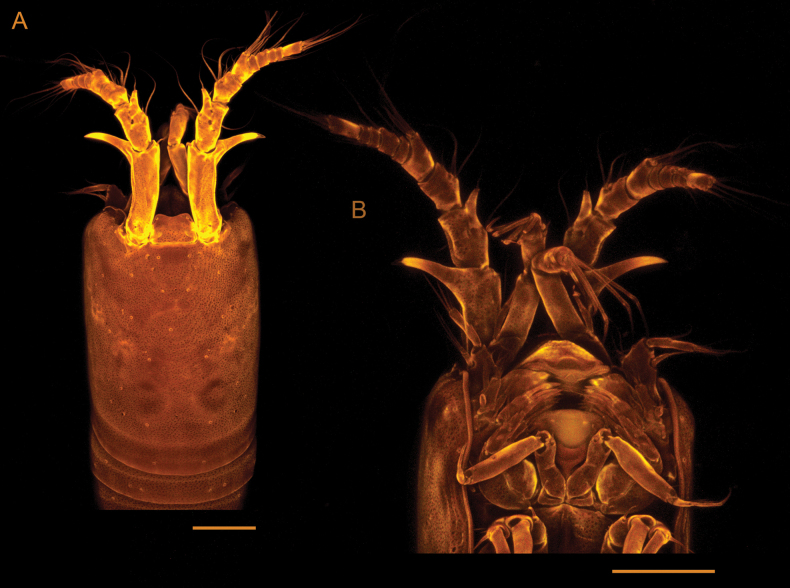
*Tetragonicepsbermudensis* sp. nov., female. Confocal laser scanning microscopy images **A** cephalothorax, in dorsal view **B** anterior part of cephalothorax and associated appendages, in ventral view. Scale bar: 50 µm.

**Female** (Figs 3, 4). Total body length from base of rostrum to end of caudal rami approximately 750 µm. Body slender, subcylindrical, with no demarcation between metasome and urosome (Fig. 3), transparent when alive. Body surface smooth, with sensilla pattern as figured (Figs 3A, B, 4). Body with 9 somites (prosome with cephalosome and P1-bearing somite fused, forming a cephalothorax, and with P_2_ to P_4_ bearing somites; urosome with P_5_-bearing somite, genital somite, 3 free urosomites, and anal somite with caudal rami). Posterior margin of body somites 4–8 with serrate hyaline frills.

**Figure 5.**
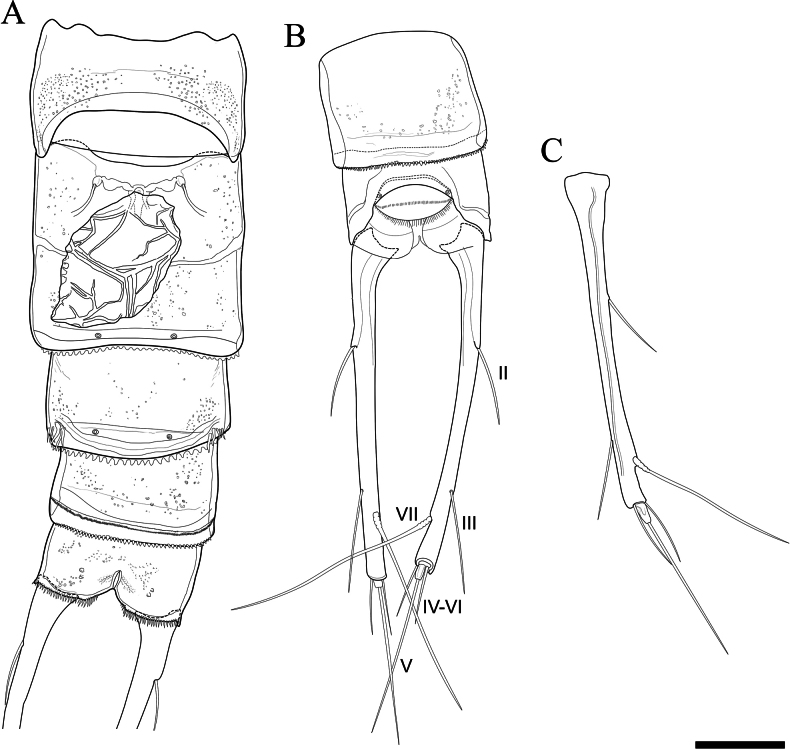
*Tetragonicepsbermudensis* sp. nov., female **A** urosome, in ventral view, showing medial egg sac **B** last two abdominal somites with caudal rami, in dorsal view **C** caudal ramus, in lateral view. Scale bar: 50 µm.

Urosome (Figs 3A–C, 5A, B) 5-segmented, comprising P_5_-bearing somite, genital double-somite, 2 free abdominal somites, and anal somite. Genital field located medially, on anterior half of genital double-somite, with small median copulatory pore and paired genital pores laterally (Fig. 5A). Anal operculum convex, with a row of fine spinules on distal margin (Fig. 5B).

Caudal rami (Figs 3A–C, 5B, C) approximately 10 times as long as wide at the widest portion (proximal end). Each ramus with 6 setae. Seta I completely reduced. Seta II laterally within the proximal half of each ramus. Seta III of similar length as seta II and located laterally within the terminal 20% of each ramus. Dorsal seta VII longer than seta V, multi-articulated. Setae IV–VI arising apically, with IV and VI reduced in size and <30% the length of seta V.

**Figure 6.**
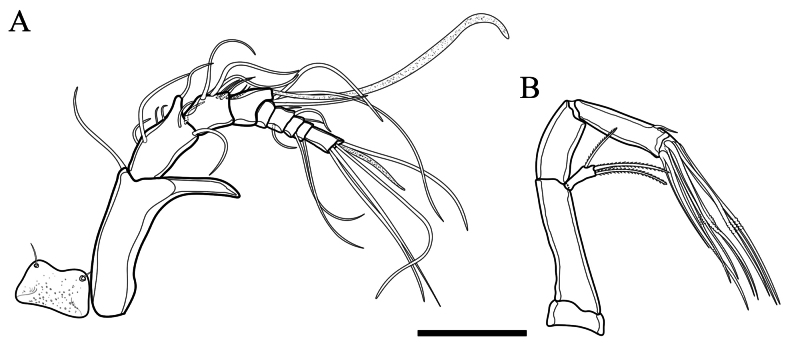
*Tetragonicepsbermudensis* sp. nov., female **A** rostrum and antennule **B** antenna. Scale bar: 50 µm.

Rostrum (Fig. 6A) rounded, symmetrical, and distally concave; it bears 2 sensilla located near the outer margin.

Antennule (Fig. 6A) 9-segmented. First segment elongate, with dentiform projection at outer distal corner. Aesthetascs on fourth and ninth segment. Armature formula 1-[1], 2-[8], 3-[8], 4-[2+ae], 5-[1], 6-[3], 7-[2], 8-[1], 9-[4+ae].

Antenna (Fig. 6B) with small, rectangular coxa and with long basis. Exopodite 1-segmented, with 2 long bipinnate setae apically and 1 shorter unipinnate seta laterally. Endopod 2-segmented. First endopodal segment unarmed. Second endopodal segment armed with 8 geniculate setae arising apically, the outer of which is basally fused with a slender seta, and armed with a small spinule on inner margin.

**Figure 7.**
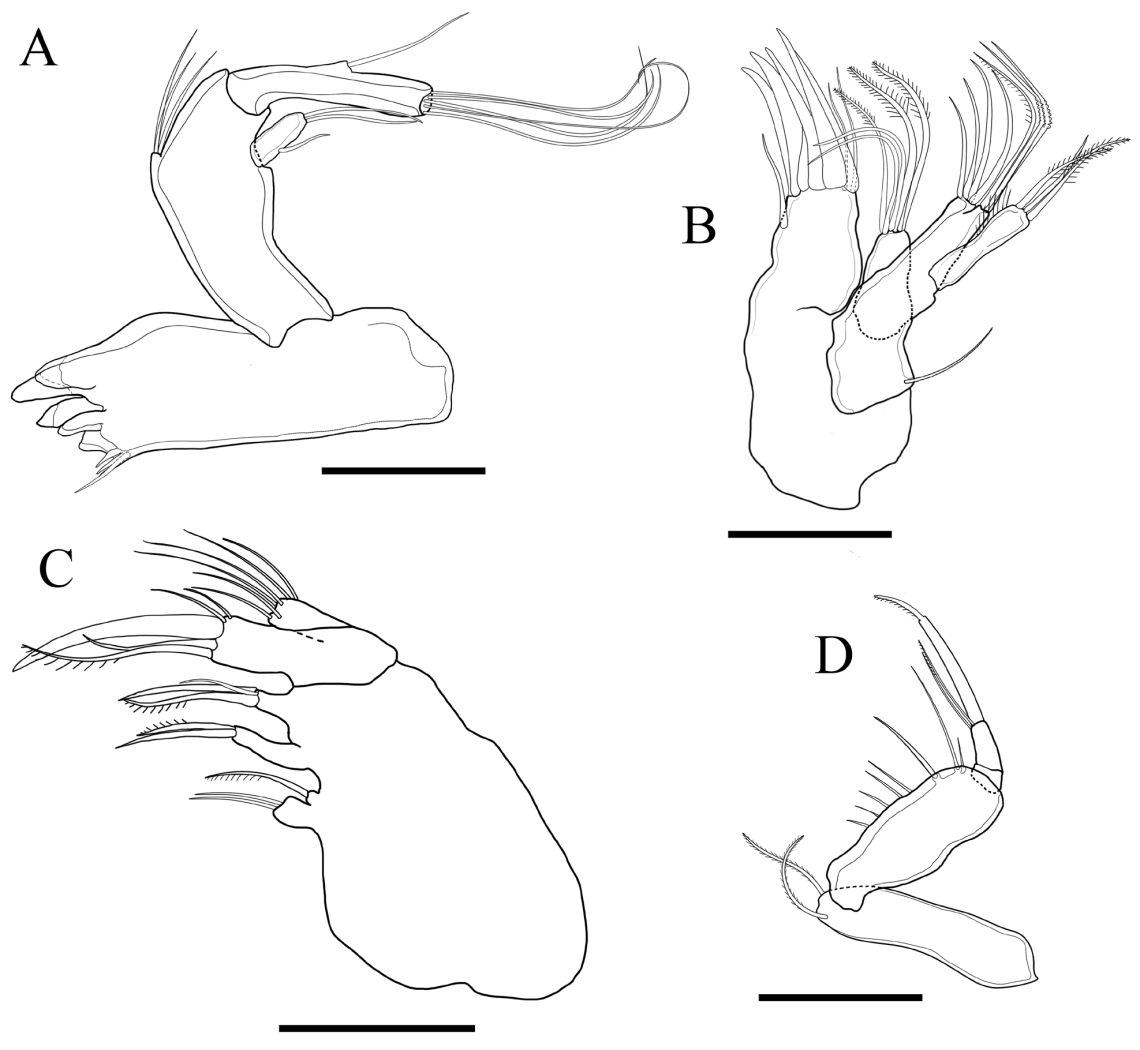
*Tetragonicepsbermudensis* sp. nov., female **A** mandible **B** maxillule **C** maxilla **D** maxilliped. Scale bars: 50 µm.

Mandible (Fig. 7A) with large sclerotised gnathobase. *Pars incisiva* with several acute teeth and several accessory spinules and dentations. Basis with 3 setae. Exopod short, 1-segmented, with 1 small outer seta and 2 terminal setae. Endopod 1-segmented, with 1 lateral and 5 terminal setae.

Maxillule (Fig. 7B) with praecoxa with arthrite armed with 6 distal spines, 2 bare terminal setae, and 1 subterminal bare seta. Coxal endite with 2 bare and 3 bipinnate terminal setae. Coxal epipodite represented by 1 bare seta. Basis with 4 bare, 1 unipinnate, and 2 geniculate terminal setae. Endopodite absent. Exopodite with 1 simple and 2 bipennate setae, and a row of long inner setules.

Maxilla (Fig. 7C), syncoxa with 3 endites, the first (proximal) bilobed, proximal lobe with two, distal lobe very reduced and armed with 1 seta. Second endite with 1 unipinnate and 1 bare seta. Third endite with 1 unipinnate and 2 bare setae, one shorter than the others. Allobasis with a claw-like spine, 1 long unipinnate seta, 1 long bare seta, and 2 short bare setae. Endopodite 1-segmented, with 6 terminal setae.

Maxilliped (Fig. 7D) subchelate. Syncoxa with 2 pinnate setae distally. Basis showing the following proximo-distally: a row of several spinules, a long spine, a long seta, and a distal spinule. Endopodite 2-segmented, second segment with 1 terminal claw, 1 terminal unipinnate seta, and 1 subterminal bare seta.

**Figure 8.**
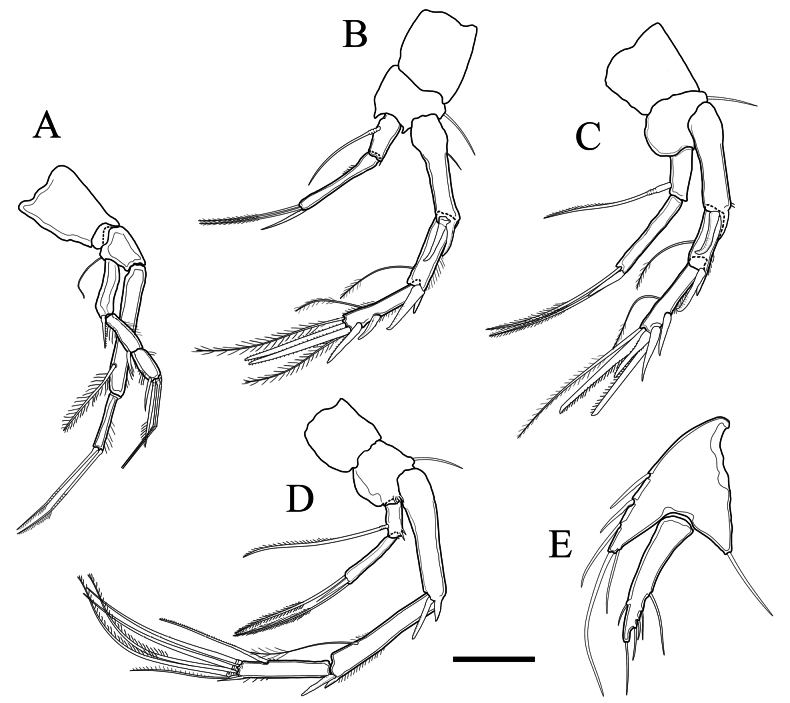
*Tetragonicepsbermudensis* sp. nov., female pereiopods **A** P_1_**B** P_2_**C** P_3_**D** P_4_**E** P_5_. Scale bar: 50 µm.

P_1_ (Fig. 8A) coxa bare, basis with 1 bare outer seta. Endopodite 2-segmented, exopodite 3-segmented. First endopodal segment with outer setules, and 1 large inner bipinnate seta. Second endopodal segment with outer setules, and 2 geniculate unipinnate setae distally. First and second exopodal segments with 1 outer spine; first segment without, second segment with inner and outer setules. Third exopodal segment with 1 outer spine, 2 geniculate setae and 1 unipinnate seta distally, and both inner and outer margin setules. Setal formula listed in Table 1.

**Table 1.** Setal formula for pereiopods P_1_–P_4_ of *Tetragonicepsbermudensis* sp. nov.

Leg	Exopodite	Endopodite
P1	0.0.022	1.020
P2	0.1.133	1.021
P3	0.1.133	1.021
P4	0.1.321	1.021

P_2_ (Fig. 8B) coxa bare, basis with 1 bare outer seta and armed with 2 short spinules surrounding the endopodite. Endopodite 2-segmented, exopodite 3-segmented. First endopodal segment with 1 large inner seta. Second endopodal segment with inner setules, 1 outer spine and 2 bipinnate setae distally. First exopodal segment with a distal spinose outgrowth, 1 large outer spine, and an outer spinule. Second exopodal segment with a distal spinose outgrowth, 1 large outer spine, 1 pinnate inner seta, and a row of outer margin spinules. Third exopodal segment with 3 outer spines, 1 pinnate inner seta, 1 outer apical spine, 2 distal bipinnate setae, and rows of both inner and outer margin spinules. Setal formula listed in Table 1.

P_3_ (Fig. 8C) coxa bare, basis with 1 bare outer seta. Endopodite 2-segmented, exopodite 3-segmented. First endopodal segment with 1 large inner seta. Second endopodal segment with 1 distal outer spine and 2 bipinnate setae distally. First exopodal segment with a distal spinose outgrowth and 1 large outer spine. Second exopodal segment with a distal spinose outgrowth and 1 large outer spine, 1 pinnate inner seta, and a row of outer spinules. Third exopodal segment with 3 large outer spines, 1 pinnate inner seta, 1 subapical bipinnate seta, 2 distal unipinnate setae, and a row of outer margin spinules. Setal formula listed in Table 1.

P_4_ (Fig. 8D) coxa bare, basis with 1 bare outer seta and armed with short spinules surrounding the endopodite. Endopodite 2-segmented, exopodite 3-segmented. First endopodal segment with 1 inner unipinnate seta and short distal spinules. Second endopodal segment with 1 outer spine and 2 bipinnate setae distally. Exopodite approximately 3 times as long as the endopodite. First exopodal segment with a distal spinose outgrowth and 1 large outer spine. Second exopodal segment with a distal spinose outgrowth and 1 large outer spine, 1 pinnate inner seta, and rows of both inner and outer spinules. Third exopodal segment with 1 small outer spine, 1 unipinnate inner seta, 2 bare setae subapically, 3 unipinnate setae distally, and rows of both inner and outer spinules. Setal formula listed in Table 1.

P_5_ (Fig. 8E), baseoendopodite very pronounced, armed with 1 outer basal seta; endopodal lobe with 1 long, bare seta apically and 1 small seta subapically, and with 3 setae on the inner margin. Exopodite pronounced, elongate, with 6 setae.

P_6_ (Fig. 5A) each with rudimentary lobe carrying 2 bare setae, fused to basal plate.

### ﻿Phylogenetic results

**Figure 9.**
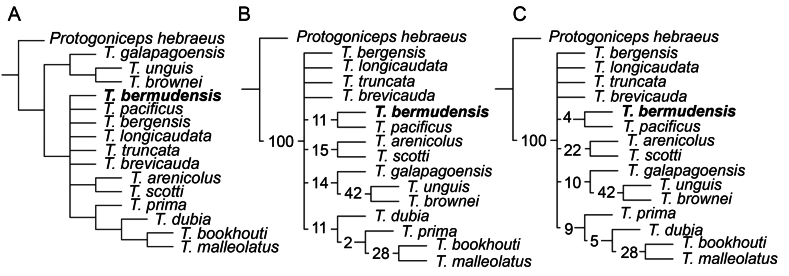
Maximum parsimony-based analyses of the genus *Tetragoniceps*, showing the recovered placements of *T.bermudensis* sp. nov. (in bold) **A** strict consensus of four most parsimonious trees based on equal weights (EW) analysis **B** standard bootstrap analysis using the same settings as in (**A**) and 100 replicates **C** jackknifing analysis using the same settings as in (**A**), with 100 replicates and removal probability of 36. For each resampling analysis, numbers at nodes denote respective support values.

Our MP strict consensus tree yielded a topology in which a monophyletic group, comprising *T.galapagoensis* plus a clade uniting *T.unguis* and *T.brownei* is the sister-taxon of all other species of *Tetragoniceps* (Fig. 9A). *Tetragonicepsbermudensis***sp. nov.** was recovered in a polytomy with *T.bergensis*, *T.pacificus*, *T.brevicauda*, *T.truncata*, *T.longicaudata*, and the following clades: one consisting of *T.prima*, *T.dubia*, *T.bookhouti*, and *T.malleolatus*; and one comprising *T.arenicolus* and *T.scotti* (Fig. 9A).

Resampling support values for both bootstrapping and jackknifing were found to be low for all recovered ingroup nodes (<50). All individual node support values under bootstrapping and jackknifing are presented in Fig. 9B and Fig. 9C, respectively. Under both bootstrapping (Fig. 9B) and jackknifing (Fig. 9C) analyses, which yielded identical topologies, *T.bermudensis* sp. nov. was recovered as the sister taxon of *T.pacificus* with low support values (<12). The clade comprising *T.prima*, *T.dubia*, *T.bookhouti*, and *T.malleolatus*, that comprising *T.arenicolus* and *T.scotti*, and that comprising *T.galapagoensis*, *T.unguis*, and *T.brownei* were found to be robust to both bootstrapping and jackknifing, albeit with low support and minor internal changes in topology (Fig. 9B, C). However, in both resampling analyses the *T.galapagoensis* + (*T.unguis* + *T.brownei*) clade collapsed into a polytomy with the other lineages within the genus, instead of being recovered as their sister-taxon (Fig. 9B, C).

## ﻿Discussion

The new species can be attributed to *Tetragoniceps* based on its first antennular segment with a diagnostic dentiform and laterally pointing projection at the posterodistal corner, which is absent in other members of Tetragonicipitidae (Coull 1973; Fiers 1995; Fiers and de Troch 2000; Gómez and Morales-Serna 2015).

*Tetragonicepsbermudensis* sp. nov. is morphologically distinct from all species of *Tetragoniceps* listed in the most recent keys (see Coull 1973; Wells 2007). Among the taxa included by Coull (1973), *T.bermudensis* sp. nov. can be distinguished from *T.bookhouti* (see Coull 1971), *T.malleolatus* (see Brady 1880), and *T.dubia* (see Thompson and Scott 1903) by the morphology of its P_5_, where the baseoendopodite and exopodite are not fused into a single plate on each side of the body. *Tetragonicepsbermudensis* sp. nov. also differs from *T.longicaudata* (see Nicholls 1940), *T.arenicolus* (see Khrishnaswamy 1957), *T.scotti* (see Sars 1911), *T.brownei* (see Wells 1967), *T.truncata* (see Nicholls 1940), and *T.brevicauda* (see Nicholls 1940) in the length of its caudal rami, which are approximately 10 times longer than wide. This character is also absent in all species described after the publication of Coull (1973), including *T.pacificus* (see Burgess 1998), *T.unguis* (see Wells and Rao 1987), *T.galapagoensis* (see Mielke 1989), and *T.santacruzensis* (see Mielke 1997), but is shared with *T.bergensis* (see Por 1965). However, the caudal rami of *T.bermudensis* sp. nov. differ from those of *T.bergensis* by having a significantly more elongate dorsal seta, longer than the terminal setae. *Tetragonicepsbermudensis* sp. nov. can also be readily distinguished from *T.bergensis* by its lack of a conspicuous dorsal dentiform projection on the cephalothorax [cf. Por 1965: fig. 23] and the diagnostic setal formula of its pereiopods P_1_–P_4_ (Table 1). The two species also differ in their pattern of setal ornamentation on P_5_: the exopodite of *T.bergensis* bears five instead of six setae as in *T.bermudensis* sp. nov., and its baseoendopodite has fewer (4) and shorter setae on its outer margin compared to the Bermudian species. Based on these data we provide an updated key to the species of *Tetragoniceps*, including all new species described after Coull (1973) for which the females are known:

### ﻿Updated key to the females of the species of *Tetragoniceps*, after Coull (1973)

**Table:** 

1	Medially pointing dentiform projection on the first segment of A_1_	***T.prima* (Coull, 1971)**
–	Distally pointing dentiform projection on the first segment of A_1_	**2**
2	P_5_ fused into single plate each side	**3**
–	P_5_ not fused into single plate each side	**5**
3	Exp. of A_2_ with 3 setae	**4**
–	Exp. of A_2_ with 2 setae	***T.bookhouti* Coull, 1971**
4	Exp. of P_5_ with only 1 well-developed seta	***T.malleolatus* Brady, 1880**
–	Exp. of P_5_ with 4 well-developed setae	***T.dubia* Thompson & A. Scott, 1903**
5	Caudal rami approximately 3 times as long as wide 6 Caudal rami at most 2 times as long as wide	**8**
–	Caudal rami approximately 10 times as long as wide	**10**
6	Middle segment of P_3_ and P_4_ exps with inner seta	**7**
–	Middle segment of P_3_ and P_4_ exps without inner seta	**8**
7	Exp. P_5_ with 3 setae; last segment exp. P_3_ with 3 outer setae; exp. A_2_ with 2 setae	***T.arenicolus* Krishnaswamy, 1957**
–	Exp. P_5_ with 6 setae; last segment exp. P_3_ with 2 outer setae; exp. A_2_ with 3 setae	**13**
8	First segment enp. P_4_ with large inner seta; exp. P_5_ with 4 setae	**9**
–	First segment enp. P_4_ without large inner seta; exp. P_5_ with 5 setae	***T.longicaudata* Nicholls, 1940**
9	Benp. P_5_ with 6 setae	***T.galapagoensis* Mielke, 1989**
–	Benp. P_5_ with 5 setae	***T.unguis* Wells & Rao, 1987**
10	Cephalothorax with dorsal dentiform projection	***T.bergensis* Por, 1965**
–	Cephalothorax without dorsal dentiform projection	***T.bermudensis* sp. nov.**
11	Exp. P_5_ with 6 setae	**12**
–	Exp. P_5_ with 4 setae; caudal rami with prominent dorsal keel; dentiform projection A_1_ small	***T.brownei* Wells, 1967**
12	Caudal rami truncate, broad at somitic attachment, rapidly compressed (i.e., tapering)	***T.truncata* Nicholls, 1940**
–	Caudal rami normal, gradually tapering	***T.brevicauda* Nicholls, 1940**
13	Last segment exp. P_3_ with one outer seta, last segment enp. P_3_ with one outer seta; 4 setae on benp. of P_5_	***T.scotti* Sars, 1911**
–	Last segment exp. P_3_ with two outer setae, last segment enp. P_3_ with one inner seta; 6 setae on benp. of P_5_	***T.pacificus* Burgess, 1998**

Salient female characters of *Tetragoniceps* (Coull 1973) offer grounds for a broader morphological comparison of *T.bermudensis* sp. nov. and other members of the genus (Table 2). The P_2_–P_4_ endopods of *T.bermudensis* sp. nov. and a group of “Indo-Pacific” species (*T.unguis*, *T.galapagoensis*, and *T.pacificus*) share the same setal formula (1.021): another species from the Indian Ocean, *T.brownei*, has a similar P_2_–P_4_ endopodal formula of 1.020 (Table 2). In addition, *T.unguis*, *T.galapagoensis*, and *T.pacificus* share with *T.bermudensis* sp. nov. the presence of three setae on the exopod of A_2_, and a P_5_ with distinct baseoendopodite and exopodite; furthermore, *T.galapagoensis* has in common with *T.bermudensis* sp. nov. a baseoendopodite of P_5_ armed with six setae (Table 2). Even closer similarities in setal armatures occur between *T.bermudensis* sp. nov. and *T.pacificus*, which also shares with the new taxon the presence of six setae on the exopodite of P_5_. Since both *T.pacificus* and *T.bermudensis* sp. nov. have a 9-segmented A_1_, the only salient characters established by Coull (1973) for which *T.pacificus* differs from the new Bermudian taxon are the length:width ratio of the caudal rami and the setal armatures of segments 1 and 3 in the exopods of P_2_–P_4_ (Table 2).

**Table 2.** Genus *Tetragoniceps*, updated summary of salient female morphological characters after Coull (1973). Abbreviations: exp., exopodite; enp., endopodite; benp., baseoendopodite. Species for which males are known are marked by an asterisk.

Species	A_1_, No. of segments	No. setae exp. A_2_	P_5_: benp. and exp.	No. setae P_5_ (benp. – exp.)	Caudal rami: approx. length/width	Setal formulae P_2_ (exp. – enp.)	Setal formulae P_3_ (exp. – enp.)	Setal formulae P_4_ (exp. – enp.)
*T.malleolatus* Brady, 1880	8	3	Confluent	4(5) – 1	?	unknown	0.1.022 – ?.121	0.1.221 – 1.120
*T.dubia* Thompson et al., 1903	8	3	Confluent	5 – 4	1:1	unknown	unknown	1.1.322 – 1.020
*T.brevicauda* Scott, 1900	9	3	Distinct	5 – 6	1.5:1	unknown	unknown	unknown
*T.scotti* Sars, 1911*	9	3	Distinct	4 – 6	3:1	unknown	1.1.122 – 1.120	1.1.123 – 1.120
*T.truncata* Nicholls, 1940	9	3	Distinct	4(5) – 6	1:1	1.0.023 – 1.120	1.0.023 –1.120	1.1.223 – 1.120
*T.longicaudata* Nicholls, 1940*	9	3	Distinct	3 – 5	3:1	1.0.023 – 1.120	1.0.023 – 1.120	1.1.123 – 1.020
*T.arenicolus* Khrishnaswamy, 1957	8	2	Distinct	4 – 3	3:1	1.1.023 – 1.020	? 1.023 – unknown	1.1.123 – 1.121
*T.bergensis* Por, 1965*	9	3	Distinct	4 – 5	10:1	0.1.222 – 1.120	0.1.221 – 1.120	0.1.223 – 1.120
*T.brownei* Wells, 1967*	8	3	Distinct	5 – 4	2:1	1.0.022 – 1.020	1.0.221 – 1.020	1.0.221 – 1.020
*T.prima* (Coull, 1971)*	8	3	Distinct	5 – 4	4:1	0.1.123 – 1.121	0.1.223 – 1.121	0.1.223 – 1.121
*T.bookhouti* Coull, 1971	8	2	Confluent	4(5) – 4	4:1	0.1.122 – 1.120	0.1.122 – 1.120	0.1.122 – 1.120
*T.unguis* Wells & Rao, 1987*	8	3	Distinct	5 – 4	3:1	1.0.022 – 1.021	1.0.021 – 1.021	1.0.221 – 1.021
*T.galapagoensis* Mielke, 1989*	8	3	Distinct	6 – 4	2.6:1	1.0.023 – 1.021	1.0.022 – 1.021	1.0.321 – 1.021
*T.santacruzensis* Mielke, 1997*	Unknown	Unknown	Unknown	Unknown	Unknown	Unknown	Unknown	Unknown
*T.pacificus* Burgess, 1998	9	3	Distinct	6 – 6	2.5:1	1.1.123 – 1.021	1.1.222 – 1.021	1.1.322 – 1.021
*T.bermudensis* sp. nov.	9	3	Distinct	6 – 6	10:1	0.1.133 – 1.021	0.1.133 – 1.021	0.1.321 – 1.021

Among *Tetragoniceps*, caudal rami approximately 10 times longer than wide are only known in the newly described Bermudian species and in *T.bergensis*, reported from Norwegian waters (Por 1965). Three setae on the exopod of A_2_ and a P_5_ with distinct baseoendopodite and exopodite, as found in *T.bermudensis* sp. nov., also occur in a broader set of North Atlantic species (*T.brevicauda*, *T.scotti*, *T.truncata*, *T.longicaudata*, and *T.bergensis*) which share with *T.bermudensis* sp. nov. a 9-segmented A_1_; the latter character is not found in “Indo-Pacific” *Tetragoniceps*, except for *T.pacificus* (Table 2).

These partly overlapping similarities of *T.bermudensis* sp. nov. with Pacific congeneric species on the one hand, and with North Atlantic ones the other, suggest that the new species may combine plesiomorphic characters retained by distinct, geographically widespread lineages of *Tetragoniceps*. This hypothesis is in accord with the recurrent archaisms expressed by Bermuda’s endemic anchialine cave faunas (Sket and Iliffe 1980; Hart et al. 1985; Iliffe and Kornicker 2009) and suggests that *T.bermudensis* sp. nov. may record an early-diverging taxon within the genus, or at least relative to its other Atlantic species.

Our MP consensus tree (Fig. 9A) is consistent with such a relatively basal placement. The only group of *Tetragoniceps* species recovered as more basal than the polytomy encompassing *T.bermudensis* sp. nov. is the clade formed by *T.galapagoensis*, *T.brownei*, and *T.unguis*, of which *T.galapagoensis* represents the earliest diverging member (Fig. 9A). This topology suggests that salient traits uniting *T.bermudensis* sp. nov. with *T.galapagoensis* and/or *Protogoniceps*, notably including a 9-segmented A_1_, three setae on the exopodite of A_2_, and distinct (non-confluent) baseoendopodite and exopodite of P_5_, are plesiomorphic for *Tetragoniceps*. This scenario is consistent with these same character states being widely retained among species in the genus (Table 2, Suppl. material 2). By contrast, confluence of the baseoendopodite and exopodite of P_5_ only occurs in the monophyletic group formed by *T.dubia*, *T.bookhouti*, and *T.malleolatus* and represents a probable synapomorphy of this clade (Fig. 9, Suppl. material 2). The other recovered clades shown in Fig. 9 are not united by any strong candidate synapomorphies, even though their members share multiple overlapping similarities in their setal armatures (Table 2, Suppl. material 2). However, these phylogenetic results must be considered preliminary given the few salient characters available for *Tetragoniceps*, the low resampling support values of our recovered topology (Fig. 9), and the lability of morphological traits within the genus (Table 2). Almost no genetic data is presently available for Tetragonicipitidae (Kim et al. 2023), and additional molecular evidence is necessary to build a robust phylogeny of the family.

*Tetragonicepsbermudensis* sp. nov. represents the first record of *Tetragoniceps* in Bermuda, the first known anchialine species in the genus, and the second record of an anchialine species of Tetragonicipitidae globally (Karanovic et al. 2001). The new taxon brings the total of anchialine copepod species in Bermuda to 22, distributed across 16 genera. The single known, geographically localised occurrence of *T.bermudensis* sp. nov. suggests a correspondingly limited area and a probable endemic status, consistent with the high degree of endemism typical of Bermuda’s cave-dwelling fauna (Iliffe and Calderón-Gutiérrez 2021). Although Roadside cave is in a relatively undisturbed area, persistent threats include urban development, vandalism, dumping, littering and pollution, and sediment disturbance due to unlawful access by humans and domesticated animals (Mammola et al. 2019; Iliffe and Calderón-Gutiérrez 2021). These risks emphasize the need for formal protection of Roadside cave, and for robust enforcement of existing measures (Glasspool 2014) for the safeguard of Bermuda’s anchialine fauna.

## Supplementary Material

XML Treatment for Tetragoniceps

XML Treatment for Tetragoniceps bermudensis

## ﻿Additional information

### Author ORCIDs

Giovanni Mussini https://orcid.org/0009-0002-1093-5865

Sahar Khodami https://orcid.org/0000-0002-7944-4004

Terue C. Kihara https://orcid.org/0000-0002-7292-251X

Pedro Martinez Arbizu https://orcid.org/0000-0002-0891-1154

Leocadio Blanco-Bercial https://orcid.org/0000-0003-0658-7183

### Data availability

All of the data that support the findings of this study are available in the main text or Supplementary Information.

This is publication number 75 that uses data from the Senckenberg am Meer Confocal Laserscanning Microscope Facility (SGN-SaM-cLSM): additional digital data relating to this publication is available in the Zenodo repository with the following DOI: https://doi.org/10.5281/zenodo.14920270.

## Supplementary materials

Supplementary material 1 Confocal laser scanning microscopy (CLSM) settings used for imaging the specimen Data type docx https://binary.pensoft.net/file/1333702This dataset is made available under the Open Database License (http://opendatacommons.org/licenses/odbl/1.0/). The Open Database License (ODbL) is a license agreement intended to allow users to freely share, modify, and use this Dataset while maintaining this same freedom for others, provided that the original source and author(s) are credited.Giovanni Mussini, Yuuki J. Niimi, Sahar Khodami, Terue C. Kihara, Pedro Martinez Arbizu, Leocadio Blanco-Bercial

Supplementary material 2 Phylogenetic dataset Data type nexus https://binary.pensoft.net/file/1333703This dataset is made available under the Open Database License (http://opendatacommons.org/licenses/odbl/1.0/). The Open Database License (ODbL) is a license agreement intended to allow users to freely share, modify, and use this Dataset while maintaining this same freedom for others, provided that the original source and author(s) are credited.Giovanni Mussini, Yuuki J. Niimi, Sahar Khodami, Terue C. Kihara, Pedro Martinez Arbizu, Leocadio Blanco-Bercial
