# Supplementary material for: ﻿A new species of Tetragoniceps Brady, 1880 (Copepoda, Harpacticoida, Tetragonicipitidae) from an anchialine cave in Bermuda, with an updated key to the species of the genus
Source: Zookeys. 2025 May 20;1239:1–19. doi: 10.3897/zookeys.1239.144436 (PMC12117344; doi:10.3897/zookeys.1239.144436)
Supplement: Supplementary material 1 — Confocal laser scanning microscopy (CLSM) settings used for imaging the specimen [file zookeys-1239-001_article-144436__-s001.docx]

SUPPLEMENTARY TABLE 1. Confocal laser scanning microscopy (CLSM) settings used for imaging the specimen. Ch1 and Ch2 = detection channels 1 and 2, PMT1 and PMT2 = Photomultiplier tube 1 and 2.

|  |  | Fig.3A | Fig.3B | Fig. 3C | Fig. 4A | Fig. 4B |
| --- | --- | --- | --- | --- | --- | --- |
| Objective |  | HCX PL APO CS 10.0x 0.40 DRY UV | | | HCX APO U-V-I 40.0 x0.75 DRY UV | |
| Numerical aperture |  | 0.40 | | | 0.75 | |
| Immersion |  | No immersion - air | | | | |
| Excitation wavelength |  | 561 nm | | | | |
| Laser intensity |  | 80% | | | | |
| Excitation beam splitter |  | Double dichroic filter 488/561 | | | | |
| Detected emission wavelength | Ch1: | 570 – 621 nm | | | 570 – 629 nm | |
|  | Ch2: | 621 – 717 nm | | | 629 – 717 nm | |
| Detector gain | PMT1: | 685.0 V | 642.0 | 685 V | 685 V | 736 V |
|  | PMT2: | 649.0 V | 595.0 | 649 V | 649 V | 714 V |
| Amplitude offset | PMT1: | -1.7 % | | | | |
|  | PMT2: | -0.8 % | | | | |
| Electronic zoom |  | 1.7 X | | | 1.0 X | |
| Pinhole aperture |  | 53.0 μm | | | 113.2 μm | |
| Voxel width/ height |  | 0.445 μm | | | 0.189 μm | |
| Voxel depth |  | 2.517 μm | 1.300 μm | 2.601 μm | 1.258 μm | 1.174 μm |
| Number of sections |  | 82 | 110 | 126 | 110 | 200 |
